# Postoperative Complications in Patients With Metabolic Syndrome Following Unicompartmental Knee Arthroplasty

**DOI:** 10.7759/cureus.71812

**Published:** 2024-10-18

**Authors:** Alyssa Schreiber, Theodore Quan, Philip Parel, Agustin Ameigeiras, Sean Ahmadi, Jeffrey Wang, Jeremy D Le, Sean A Tabaie

**Affiliations:** 1 Orthopaedic Surgery, George Washington University School of Medicine and Health Sciences, Washington, D.C., USA; 2 Orthopaedic Surgery, Nationwide Children’s Hospital, Columbus, USA

**Keywords:** complications, metabolic syndrome, orthopedic surgery, postoperative outcomes, unicompartmental knee arthroplasty

## Abstract

Introduction: Metabolic syndrome is associated with increased risk of postoperative complications following various orthopedic surgeries. This study looked to identify specific postoperative complications in these patients following unicompartmental knee arthroplasty (UKA).

Methods: Adult patients undergoing primary UKA were queried in the National Surgical Quality Improvement Program database from 2006-2019. Metabolic syndrome was defined by the simultaneous presence of hypertension, diabetes, and body mass index >30 kg/m^2^. Two patient groups were categorized in this study: patients with metabolic syndrome and patients without metabolic syndrome. Baseline characteristics and postoperative outcomes were compared between the two cohorts with the use of bivariate and multivariate analyses.

Results: In total, 10,557 patients underwent UKA. Of these, 9,511 patients (90.1%) did not have metabolic syndrome whereas 1,046 (9.9%) had metabolic syndrome. Following adjustment for potential confounding variables on multivariate analysis, metabolic syndrome patients had an increased risk of an extended length of stay greater than four days (OR 1.66; p=0.016) compared to patients without metabolic syndrome.

Conclusion: Metabolic syndrome was associated with an increased risk of extended length of stay greater than four days following UKA. Physicians should be prepared for this complication as well as others when discussing treatment with metabolic syndrome patients.

## Introduction

Metabolic syndrome is defined as a combination of factors that increase the risk of cardiovascular disease. The criteria for diagnosis require at least three of the following five factors: (i) increased waist circumference, (ii) elevated triglycerides (≥150 mg/dL), (iii) reduced high-density lipoprotein cholesterol (HDL-C) defined as <40 mg/dL in men and <50 mg/dL in women, (iv) high blood pressure (systolic ≥130 and/or diastolic ≥85 mmHg), and (v) elevated fasting glucose (≥100 mg/dL), and accounts for medication used to treat any of the named conditions [1].

As rates of obesity and type 2 diabetes have risen in the United States, so has the prevalence of metabolic syndrome, which has been estimated to be present in about one-quarter of the world’s population [2]. These patients have approximately a two-fold increase in cardiovascular disease and are at increased risk for other medical problems such as fatty liver disease and cancer [3]. Metabolic syndrome has such a strong correlation to comorbidities such as cardiovascular disease and kidney disease that a condition called cardiovascular-kidney and metabolic (CKM) syndrome has been identified [4]. A patient with or at risk of CKM syndrome suffers from a multitude of complications that include hypertension, obesity, atherosclerosis, and kidney failure [4].

Patients with metabolic syndrome are an important cohort to study due to this increased presence of associated diseases and thus adverse events, which becomes especially important to consider in surgical procedures. Hypertension has been shown to contribute to a greater risk of postoperative complications in patients undergoing total knee arthroplasty (TKA); Li et al. identified that patients with hypertension undergoing TKA had a greater occurrence of postoperative complications such as electrolyte disorders, acute renal failure, urinary tract infections, and respiratory failure [5]. Chronic kidney disease (CKD) has shown a similar correlation to postoperative complications in orthopedic surgeries [6]. Since metabolic syndrome can contribute to both renal and cardiovascular disease, it is an important and relevant factor to consider when analyzing a patient’s risk of surgical complications. To our knowledge, no study has looked to identify complications in unicompartmental knee arthroplasty (UKA) among these patients.

This article was previously a poster presentation at the American Academy of Orthopaedic Surgeons in March 2023. 

## Materials and methods

The American College of Surgeons (ACS)'s National Surgical Quality Improvement Program (NSQIP) database was used for this investigation from 2006 to 2019. NSQIP is a high-quality, prospectively collected surgical database that encompasses over 750 medical centers nationwide. The high-quality feature of this database stems from the fact that it excludes hospitals whose interobserver disagreement rate between clinical reviewers is greater than 5% [7]. This database provides de-identified data regarding patient demographics, comorbidities, and postoperative outcomes, which has been validated for many orthopedic procedures to date [8-10]. The study is exempt from institutional board approval.

Patient selection

Current procedural terminology (CPT) code 27446 was used to identify all patients who underwent primary UKA. Only patients with the primary procedure listed as UKA were selected for this study. Patients were excluded if their UKA was performed for a diagnosis other than osteoarthritis of the knee. Patients were also excluded from this study if they were missing baseline values, such as if their race or gender were unknown. Patients less than 18 years old were further excluded from the study. Based on prior studies, metabolic syndrome was defined by the simultaneous presence of diabetes mellitus, hypertension, and body mass index (BMI) >30 kg/m^2^ [11]. Two patient groups were identified for this study: patients with metabolic syndrome and patients without metabolic syndrome.

Data collection

Baseline characteristics collected included age, gender, race, American Society of Anesthesiologists (ASA) score, and functional and smoking status. Baseline comorbidities extracted from the database included congestive heart failure (CHF), chronic obstructive pulmonary disease (COPD), dialysis, bleeding disorder, preoperative transfusion requirement, steroid use, and dyspnea. Anesthesia type was also recorded.

Postoperative outcomes

The 30-day outcomes following UKA investigated for this study included superficial/deep surgical site infections, organ/space infections, wound dehiscence, pneumonia, pulmonary embolism, urinary tract infection, stroke, renal failure, blood transfusion requirement, deep vein thrombosis, sepsis, myocardial infarction, extended length of stay, readmission, return to the operating room, and mortality. Extended length of stay was defined as > 4 days based on the existing literature [12].

Statistical analysis

Demographic and comorbidity data were compared between the patient groups using Pearson’s Chi-squared test and analysis of variance where appropriate. Postoperative outcomes were also compared between the cohorts with Chi-squared tests. All demographic and comorbidity variables with a p-value < 0.20 in the bivariate analyses were included in a multiple logistic regression analysis to adjust for confounders [13]. Odds ratios (ORs) and 95% confidence intervals (CIs) were reported from the multivariate analysis results. Statistical significance was maintained at a p-value < 0.05. All analyses were done using IBM SPSS Statistics for Windows, Version 28.0 (Released 2021; IBM Corp., Armonk, New York, United States).

## Results

Patient demographics

Upon application of the exclusion criteria, 10,557 patients who underwent UKA were included in the analysis. A total of 9,511 patients (90.1%) did not have metabolic syndrome whereas 1,046 (9.9%) had metabolic syndrome. In comparison to the non-metabolic syndrome cohort, patients with metabolic syndrome were more likely to be male (56.8% vs 48.5%; p<0.001), Hispanic (7.0% vs 3.8%; p<0.001), and have an ASA score of III or IV (76.6% vs 36.9%; p<0.001) (Table 1). 

**Table 1:** Baseline demographics and clinical characteristics of unilateral knee arthroplasty patients according to the presence or absence of metabolic syndrome ¶Pearson’s chi-squared test; **Analysis of variance p<0.05 shows significance; Data shown as n (%), except for age which is given as mean±SD ASA, American Society of Anesthesiologists

Demographics	No Metabolic Syndrome (n=9,511), n (%)	Metabolic Syndrome (n=1,046), n (%)	P-value
Gender			< 0.001^¶^
Female	4,894 (51.5)	452 (43.2)	
Male	4,617 (48.5)	594 (56.8)	
Race			< 0.001^¶^
White	8,413 (88.5)	875 (83.7)	
Black	383 (4.0)	59 (5.6)	
Hispanic	361 (3.8)	73 (7.0)	
Native American	24 (0.3)	5 (0.5)	
Asian	292 (3.1)	28 (2.7)	
Native Hawaiian	38 (0.4)	6 (0.6)	
ASA			< 0.001^¶^
I or II	5,999 (63.1)	245 (23.4)	
III or IV	3,502 (36.9)	800 (76.6)	
Smoker	904 (9.5)	96 (9.2)	0.732^¶^
Dependent functional status	82 (0.9)	13 (1.3)	0.214^¶^
Age (years), mean±SD	64.14 (10.54)	65.35 (9.07)	< 0.001**

Patient comorbidities

Compared to patients who did not have metabolic syndrome, those with metabolic syndrome were more likely to have other medical comorbidities, including COPD (4.6% vs 2.9%; p=0.002), current dialysis (0.5% vs 0.1%; p=0.004), bleeding disorder (2.8% vs 1.7%; p=0.016), and dyspnea on moderate exertion (6.9% vs 3.9%; p<0.001). Metabolic syndrome patients were also more likely to undergo general anesthesia (55.8% vs 48.7%; p=0.006) (Table 2).

**Table 2:** Comorbidities and intraoperative variables among unilateral knee arthroplasty patients ¶Pearson’s chi-squared test p<0.05 shows significance CHF, congestive heart failure; COPD, chronic obstructive pulmonary disease; MAC, monitored anesthetic care.

Comorbidities	No Metabolic Syndrome (n=9,511), n (%)	Metabolic Syndrome (n=1,046), n (%)	p-value^ ¶^
CHF	17 (0.2)	4 (0.4)	0.161
COPD	274 (2.9)	48 (4.6)	0.002
Dialysis	11 (0.1)	5 (0.5)	0.004
Bleeding disorder	164 (1.7)	29 (2.8)	0.016
Preoperative blood transfusion	1 (0.0)	0 (0.0)	0.740
Chronic steroid use	186 (2.0)	25 (2.4)	0.341
Dyspnea			< 0.001
Moderate exertion	371 (3.9)	72 (6.9)	
At rest	10 (0.1)	3 (0.3)	
Anesthesia type			0.006
General	4,533 (48.7)	573 (55.8)	
Neuraxial	3,391 (36.4)	327 (31.8)	
Regional	413 (4.4)	38 (3.7)	
MAC	942 (10.1)	88 (8.6)	

​​​Patient outcomes

On bivariate analysis, when compared to non-metabolic syndrome patients, those with this condition were more likely to experience pneumonia (p=0.043), urinary tract infections (p=0.029), sepsis (p=0.011), extended length of stay > 4 days (p<0.001), and hospital readmission (p=0.016) (Table 3). Following adjustment for potential confounding variables on multivariate regression models, metabolic syndrome patients no longer had an increased risk for any postoperative complication except for an extended length of stay > 4 days (OR 1.663; 95% CI 1.100 to 2.515; p=0.016) (Table 4).

**Table 3:** Bivariate analysis of postoperative complications for unilateral knee arthroplasty patients ¶Pearson’s chi-squared test p<0.05 shows significance

Complications	No Metabolic Syndrome (n=9,511), n (%)	Metabolic Syndrome (n=1,046), n (%)	p-value^¶^
Superficial Surgical Site Infection	43 (0.5)	2 (0.2)	0.219
Deep Surgical Site Infection	10 (0.1)	2 (0.2)	0.433
Organ/Space Infection	19 (0.2)	3 (0.3)	0.558
Wound Dehiscence	11 (0.1)	2 (0.2)	0.508
Pneumonia	12 (0.1)	4 (0.4)	0.043
Pulmonary Embolism	18 (0.2)	2 (0.2)	0.989
Urinary Tract Infection	43 (0.5)	10 (1.0)	0.029
Stroke	3 (0.0)	0 (0.0)	0.566
Renal Failure	1 (0.0)	0 (0.0)	0.740
Transfusion Requirement	32 (0.3)	3 (0.3)	0.791
Deep Vein Thrombosis	29 (0.3)	3 (0.3)	0.919
Sepsis	13 (0.1)	5 (0.5)	0.011
Myocardial Infarction	10 (0.1)	1 (0.1)	0.928
Extended Length of Stay (>4 days)	141 (1.5)	31 (3.0)	< 0.001
Readmission	177 (2.5)	31 (3.9)	0.016
Reoperation	85 (0.9)	11 (1.1)	0.610
Death	5 (0.1)	1 (0.1)	0.579

**Table 4:** Multivariate analysis of postoperative complications of patients following unilateral knee arthroplasty p<0.05 shows significance

Metabolic Syndrome (versus No Metabolic Syndrome)	Odds Ratio	95% CI		P-Value
Pneumonia	1.891	0.557	6.412	0.307
Urinary Tract Infection	2.075	0.996	4.325	0.051
Sepsis	2.886	0.948	8.783	0.062
Extended Length of Stay (>4 days)	1.663	1.100	2.515	0.016
Readmission	1.245	0.830	1.868	0.288

## Discussion

As metabolic syndrome diagnoses rise in prevalence, it is becoming increasingly important to consider how it contributes to patient outcomes. Saklayen estimates in his report that metabolic syndrome is present in one-quarter of the world’s population [2]. It is therefore likely that orthopedic surgeons will encounter patients with metabolic syndrome, and surgeons must consider how this affects a patient’s risk of complications. For example, metabolic syndrome has a significant correlation with cardiovascular and renal disease, thus giving rise to CKM Syndrome [4]. Both cardiovascular and renal disease have been studied in orthopedic surgeries, and both contribute to postoperative complications [5,6]. Extensive research has been conducted on metabolic syndrome and the risk of postoperative complications in a variety of procedures, but no research has yet looked specifically at UKA to our knowledge.

Prior research has studied outcomes in patients with metabolic syndrome undergoing various orthopedic procedures. Cheng et al. evaluated wound complications after total hip arthroplasty (THA) and found that the risk of wound complications was four-fold higher in metabolic syndrome patients [14]. A systematic review and meta-analysis found that metabolic syndrome increases the risk of all-cause complications following TKA and THA, including cardiac complications, infection, and urinary tract infections [15]. An additional study found that metabolic syndrome patients were at an increased risk of deep vein thrombosis (DVT) following TKA and THA [16]. A review done by Bajaj et al. specifically looked at outcomes of spinal surgery on patients with metabolic syndrome and found that, overall, these patients are at a greater risk for increased length of stay following their procedure [17]. In reverse shoulder arthroplasty (RSA), Marigi et al. found that deep infection, instability, and DVT or pulmonary embolism occurred more often in patients with metabolic syndrome than in patients without this condition [18]. 

Our study found that, following adjustment for potential confounding variables, metabolic syndrome patients undergoing UKA had an extended length of stay > 4 days. On initial bivariate analysis before adjustment, metabolic syndrome patients undergoing UKA were more likely to have pneumonia, urinary tract infections, sepsis, hospital readmission, and extended length of stay > 4 days. This research adds to the literature by looking specifically at UKA, and the outcomes reflect those of similar studies looking at different operations by identifying that metabolic syndrome patients are more likely to suffer postoperative complications such as extended length of stay. This is especially true of Bajaj et al.’s research [17].

Our most significant result is that metabolic syndrome patients are at increased risk for extended length of stay > 4 days (OR 1.663; 95%CI 1.100-2.515; p=0.016). This correlates specifically with Bajaj et al.’s findings in spinal surgery. After adjustment for potential confounding variables, our study did not support a finding of increased risk of all-cause complications like that seen in Guofeng et al., nor did it support any specific postoperative complications like those found in Cheng et al. [14], Marigi et al. [15], and Song et al. [16]. However, our findings are relevant in adequately preparing patients for what to expect following UKA. Orthopedic surgeons can advise patients with metabolic syndrome that they are at an increased risk of extended length of stay following UKA, which helps both the hospital and the patient to sufficiently prepare for the surgery. Although following adjustment our specific postoperative complications were no longer significant, it is still important to keep these in mind also for adequate planning and preparation. The patients with metabolic syndrome in our study were more likely to have other medical comorbidities, including COPD, current dialysis, dyspnea on moderate exertion, and bleeding disorders. These comorbidities place patients at higher risk for poorer outcomes. Thus, it is beneficial for hospital staff to be aware of these potential postoperative complications due to the increased prevalence of comorbidities in metabolic syndrome patients.

Limitations of the study

While we were able to control for these comorbidities mentioned in multivariate analysis, any other comorbidities that were not reported in the database may have influenced the results since they were not accounted for. Additionally, the NSQIP database only records data for up to 30 days post surgery. This limits our study as only short-term complications could be included. It is therefore recommended that future studies examine any long-term complications present. The NSQIP database also does not include any data on patient-reported outcomes. Future studies could evaluate these outcomes in metabolic syndrome patients following UKA. Lastly, this study may have erroneous data entry or errors in data omission as it is a database study.

## Conclusions

The United States has seen a steady increase in rates of obesity and type 2 diabetes, both of which contribute to metabolic syndrome. As the prevalence of metabolic syndrome increases, it is important to consider the effects it can have in postoperative complications. This study sought to identify postoperative complications in patients with metabolic syndrome following UKA. Our research found that this group of patients are at an increased risk of extended length of stay > 4 days when compared to patients without metabolic syndrome. This information can help physicians to better prepare for potential complications in this prominent patient population, thus improving expectations and outcomes following UKA.
